# Terahertz Dielectric Spectroscopic Analysis of Polypropylene Aging Caused by Exposure to Ultraviolet Radiation

**DOI:** 10.3390/polym11122001

**Published:** 2019-12-03

**Authors:** Tianying Chang, Xiansheng Zhang, Hong-Liang Cui

**Affiliations:** 1 Institute of Automation, Qilu University of Technology (Shandong Academy of Sciences), Jinan 250014, China; 2 College of Instrumentation and Electrical Engineering, Jilin University, Changchun 130061, China; 3 Chongqing Institute of Green and Intelligent Technology, Chinese Academy of Sciences, Chongqing 400714, China

**Keywords:** polypropylene, ultraviolet aging, THz dielectric spectroscopy

## Abstract

Terahertz dielectric spectroscopy is shown to be an effective tool for bench-marking ultraviolet aging of polypropylene. In this, thin-film polypropylene samples exposed to standard artificial ultraviolet radiation in accelerated aging from 1 day to 30 days are closely monitored by a terahertz time-domain spectroscopy system and analyzed using an effective data processing method. It is found that the terahertz absorption of the polypropylene samples is generally weak and the terahertz dielectric constant varies slightly though discernibly during the aging process, with the refractive index decreasing slightly with increasing length of ultraviolet exposure. Nonetheless, the rate of variation of the polypropylene refractive index with increasing terahertz frequency exhibits a drastic inflection around the 15-days aging point and the rate changes suddenly from positive value to negative value. Based on this prominent and consistent THz dielectric spectroscopic feature of the aging process, an efficient judging criterion is established to distinguish the early-term, mid-term, and late-term phases as well as the degree of polypropylene ultraviolet aging, corresponding to the fundamental transformation of the polymer material from a nonpolar to a polar substance at a critical level of oxidation induced by ultraviolet radiation.

## 1. Introduction 

Polypropylene (PP), a nonpolar polymer with wide-ranging applications, is prone to degradation, embrittlement, and yellowing when it is subjected to strong illumination, heating, or other severe environmental stress. Its aging severely impacts its usability and reduces its service life. Therefore, understanding the aging mechanism and ascertaining the degree of aging of polymer materials in general and PP in particular are deemed important for polymer improvement in the application fields. There are three main aging categories, thermal [1,2,3], ultraviolet [4] and salt fog, according to the exposure conditions. With regard to aging testing, besides physical and mechanical methods [5,6], spectral tests, such as Raman [7,8], infrared [8,9], millimeter wave and microwave [10,11], and ultraviolet spectroscopies [12], are gaining popularity for their nondestructive nature. However, based on most of the above methods, the interior of the material cannot be pried into due to electromagnetic wave’s poor penetrability at high frequencies, with the exception of millimeter wave. Therefore, the material’s early stage of aging cannot be judged based on purely spectroscopic means [13]. 

Terahertz (THz) wave (with frequencies in the range of 0.1–10 THz) possesses not only lightwave’s photonic characteristics but also electromagnetic radiation’s wave characteristics [14,15]. In comparison with other parts of the electromagnetic spectrum, THz wave has stronger penetrability than infrared, better spatial resolution than microwave, and is safer and more benign than X-ray. It does not destroy the material’s molecular structure, nor affect the tester’s health, due to its low single photon energy. Therefore, THz technology is finding increasing applications in polymer nondestructive measurement and spectral analysis [16,17]. More importantly, THz spectrum can provide fingerprint spectral information related to polymer molecular structure and morphologic changes. As polymer ages, its molecular structural and morphologic changes lead to its THz spectral change. More specifically for PP, the degree of PP oxidation is increased as its aging process unfolds and results in increased polar oxygenated groups’ generation, and PP’s polarization. As THz waves are more sensitive to polar material, it can be inferred that THz technology could be an effective tool to evaluate PP’s aging. Natural aging and its cause and ramifications are scientifically interesting and practically important, but it takes a long time to run its full course and it is uncontrollable. Therefore, various artificial aging methods are adopted to simulate and accelerate the aging process to facilitate aging-related research. In this paper, an artificial ultraviolet exposure related aging procedure is carefully monitored to deal with the samples, and a terahertz time-domain spectroscopy (THz-TDS) system is utilized to measure the samples’ complex dielectric permittivity as a function of exposure/aging time, which is shown to be an efficient indicator of polymer UV aging.

This paper reports on experimental and analytical results of ultraviolet aging of PP studied by THz dielectric spectroscopy. In this, several prevalent methods to evaluate the aging of polymer material are introduced, along with their advantages and shortcomings. Based on this, we demonstrate the reason that THz technology is expected to be an effective tool to evaluate PP’s aging. The remainder of the paper is organized as follows: Section 2 describes the PP sample preparation and the setup of the THz-TDS system, Section 3 expounds the experiments and the analysis of PP’s dielectric spectroscopy in THz frequency during its ultraviolet aging, and Section 4 is devoted to the discussions about the experiment’s results, along with related conclusions.

## 2. Samples and Instrument

Ninety PP samples were prepared from the same isotatic PP sheet (purchased from Goodfellow Corporation, Coraopolis, PA, USA, with Tinuvin 770 as the amine stabilizer in the PP sheet), which could minimize the individual differences. The samples were cut into 80 × 80 mm squares, much larger than the THz-TDS system’s beam spot size. Since the sample’s THz dielectric property was measured by the THz-TDS system in advance of the input of its thickness, the thickness was measured by a micrometer with the accuracy of 10 μm. The range of the thickness is from 0.7 mm to 1.0 mm. The 90 pieces of PP samples were arranged as 30 groups with each group comprised of three samples, marked from 1 day to 30 days according to number of aging days. On the other hand, they are also classified as three groups with each group consists of 30 samples, marked as 1-1, 1-2, 1-3, 2-1, 2-2, 2-3, …30-1, 30-2, 30-3. In this sample designation scheme, the first number refers to the number of days the given sample was aged under ultraviolet radiation continuously and the second number labels a particular member of a given group of three samples that have all aged for the same number of days. In this manner, aging continuity was guaranteed without regard to individual sample differences. The group of three and multi-measurement hereinafter can validate the result’s consistency and reliability, while minimizing system errors and manual measurement errors. 

The FICO THz-TDS system from Zomega, Albany, NY, USA, was employed in this work and was described in detail elsewhere [18]. It has two running modes, broad bandwidth with 0–4 THz and 50 dB dynamic range and high dynamic range with 0–2 THz and 70 dB dynamic range, respectively. It has two modes of operation, transmission mode and reflection mode, respectively. The high dynamic mode and the transmission mode were used in this work. In this case, it features an 11-GHz frequency resolution, with average output power in the 10–100 nW range, and about 1.8 mm beam spot size.

## 3. Experiment and Analysis

### 3.1. Experimental Setup

In this work, the artificially controlled environment for ultraviolet aging is [19] an exposure cycle of 12 h, including the dehydration of 8 h under black standard temperature (BST) of 60 °C ± 3 °C and the condensation of 4 h under BST 50 °C ± 3 °C The samples undergoing the dehydration cycle were illuminated by the ultraviolet light UVA-340 with the wavelength 340 nm and the radiant emittance of 0.76 W·m^−2^·nm^−1^. The light was turned off during the 4 h condensation period that was also the spray stage. Periodic spray can quickly take out the hindered amine light stabilizer (HALS) on the sample’s surface so that HALS molecules from the interior of the sample do not have time to supplement to the surface. The above regimen, repeatedly applied, can accelerate the aging process efficiently. Under this artificial ultraviolet aging condition, empirically speaking, an exposure cycle of 12 h is approximately equivalent to the natural exposure aging of 22 days, under standard sunlight condition. On the other hand, due to the slight thickness of the tailor-made PP samples, PP material’s aging characteristics can be fully demonstrated.

**Figure 1:**
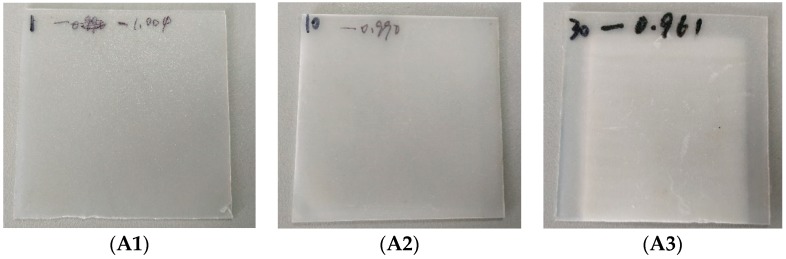
Photographs polypropylene (PP) samples at different aged stages; (**A1**). 1d aged PP sample, (**A2**). 10d aged PP sample, (**A3**). 30d aged PP sample.

After every group of three samples had gone through two exposure cycles or its integral multiple of cycles, they were taken out and detected by the THz-TDS system. Two consecutive exposure cycles is considered as one day’s exposure and the whole aging process took 30 days for the 90 samples. With the aging on-going, the PP samples’ appearance changed in a number of ways, such as surface yellowing, chalking, increased fragility, mechanical breakage, and different odors. The PP sample becomes increasingly opaque for 1-day aged, 10-days aged and 30-days aged. The 30-days aged PP sample’s aged area is obviously different from its marginal unaged area as it was covered by the holder during the ultraviolet exposure, as shown Figure 1. After 30-days aging under UV radiation, the samples all changed completely in both appearance and mechanical behavior, they become discolored and brittle, and easily fragmented when touched. Thus, there was no need to go beyond 30-days in this accelerated UV aging procedure.

### 3.2. THz Dielectric Spectroscopy of Samples

Ninety PP samples at different aged stages were detected by the THz-TDS system to obtain their respective dielectric permittivity in the THz frequency band from 0.2–2 THz. The dry atmosphere was detected as a reference signal before each sample’s detection and the corresponding sample’s thickness with the accuracy of 10 μm was inputted to calculate its dielectric constants. Every measurement was repeated 1000 times and the average was taken as the final reading. The above actions effectively eliminated the effects of the system’s instability and lowered the uncertainty of the experimental results. The samples’ THz dielectric spectroscopic data can be obtained directly from the THz-TDS system.

**Figure 2:**
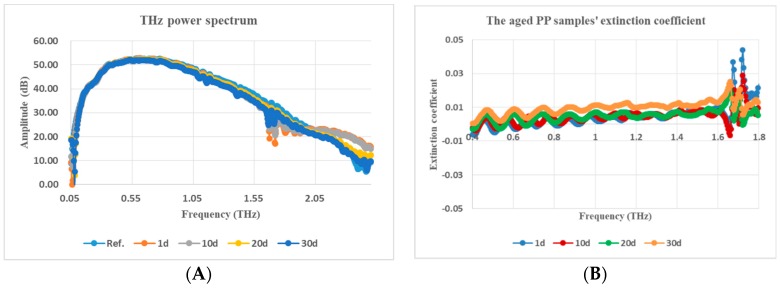
Terahertz (THz) frequency transmission spectra (**A**) and extinction coefficients (**B**) of samples during aging.

As expected, PP material’s absorption of THz waves is very weak and is validated from its THz frequency spectrum and extinction coefficient curve with THz frequency, as shown in Figure 2. Therefore, the imaginary part of its complex dielectric constant, also proportional to its extinction coefficient, cannot be used as a useful marker of aging because it always fluctuates around zero, mostly falling within the measurement accuracy of the system.

**Figure 3:**
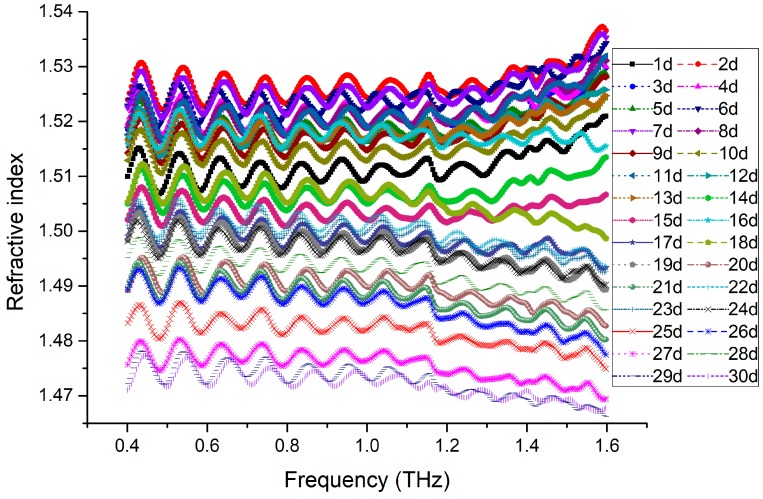
Refractive index vs. THz frequency for samples in different aged states.

**Figure 4:**
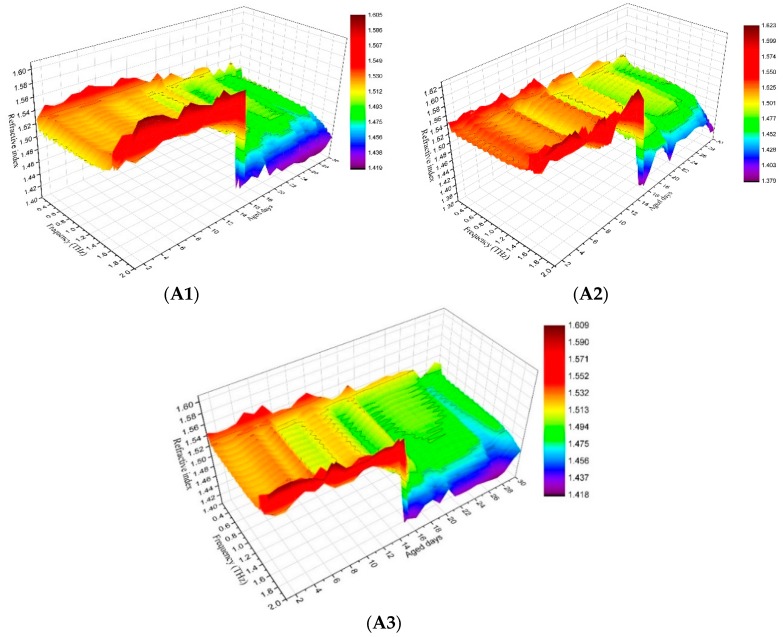
Three-dimensional diagram between refractive index, THz frequency and aged days (**A1**, **A2**, **A3** stands for -1, -2, -3 group).

**Figure 5:**
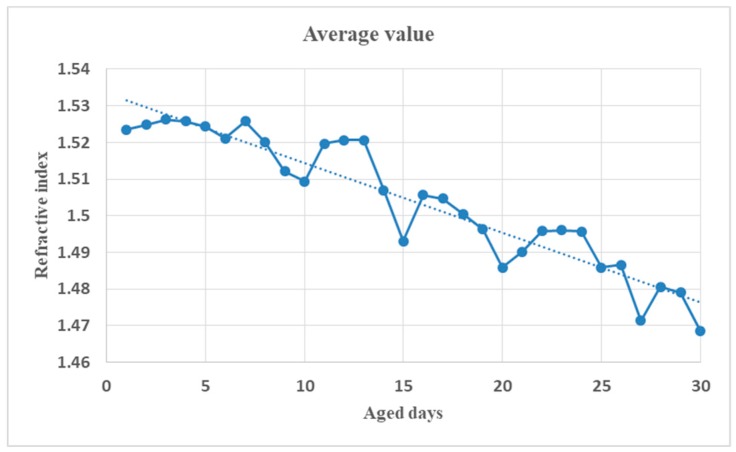
Relationship between the average value of refractive index and the aged days (The dotted straight line is its linear fitting).

**Figure 6:**
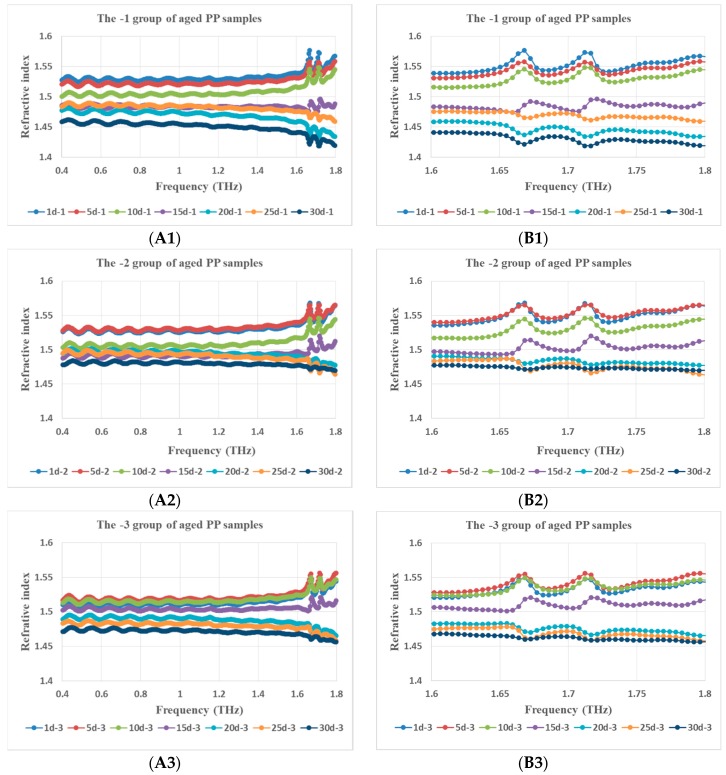
Refractive index vs. THz frequency for 3 groups of PP samples in different aged states (**A1**, **A2**, **A3** stands for -1, -2, -3 group and **B** is the enlargement of part **A**).

The real part of the complex dielectric constant of PP, that is the refractive index whose variation tendency with THz frequency for PP samples from 1 d to 30 d aged status is shown in Figure 3 and in a clearer three-dimensional (3D) diagram in Figure 4. The relationship between the average value of the refractive index in the whole THz band and the number of aged days is shown in Figure 5. Although Fabry–Perot (F–P) resonances of the refractive index curve is ubiquitous and annoying, it can be concluded that the general trend of the refractive index is that it decreased with the increasing number of aged days, the curves rise with THz frequency before 15 d aged and descend after 15 d aged. The phenomenon is reliable and stable. From Figure 4 and Figure 6, the refractive index’s variation tendency with THz frequency for 3 groups of aged PP samples were essentially uniform. 

### 3.3. Mechanism of UV Induced PP Aging

It is known that every chain link of a PP molecule has a Methyl branched chain, or more strictly speaking, contains a tertiary carbon atom, as labeled * in the following molecular Formula (1). A PP molecule can become very active if its hydrogen atom or tertiary hydrogen atom is taken off under the action of external factors and will be easily invaded by an oxygen atom to result in aging.

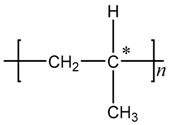
(1)

In the process of ultraviolet (UV) aging, the carbonyl (C=O) and the hydrogen peroxide (HOO) are the induction radicals of light-oxygen reaction. The carbonyl in PP can absorb UV light within the wavelength band of 260–340 nm, and the hydrogen peroxide’s absorption band can extend beyond 300 nm. The following three kinds of carbonyl can be generated in the process of light–oxygen reaction [20].

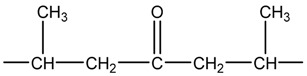
(2)

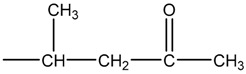
(3)

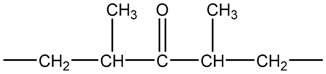
(4)

If a PP molecule containing the first carbonyl as Formula (2) is stimulated by UV light, it undergoes the Norrish type-I photochemical reaction [21,22], with free radicals generated as follows [19].

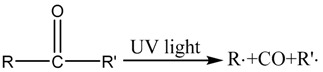
(5)

On the other hand, a PP molecule containing the second carbonyl as in Formula (3) undergoes the Norrish type-II reaction [21,22], as indicated in Formula (6) [20].

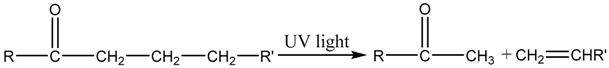
(6)

The peroxide containing peroxy-radicals in a PP molecule mainly decomposes into alcoxyl free radicals and oxyhydrogen free radical, as shown in Formula (7).

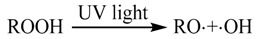
(7)

PP’s photooxidation will occur after the free radicals are generated in the photochemical reactions and will proceed according to the chain reaction mechanism of free radicals, similar to the process of thermo-oxidative aging [23].

Actually, as the aging process continues, the degree of oxidation is increased and the formation of doubly bonded oxygen is generated, as well as the formation of bonds to several atoms (C=O and O–C=O) [24]. That is to say, PP’s photooxidation accompanying the photochemical reaction could result in the transformation of the nonpolar hydrocarbon groups to polar oxygenated groups during the aging process of PP. Therefore, the degree of PP oxidation depends on the corresponding degree of its polarity, as well as its degree of aging. This chemistry could be invoked to understand the aged PP’s refractive index’s variation tendency as aging proceeds.

### 3.4. Data Processing

**Figure 7:**
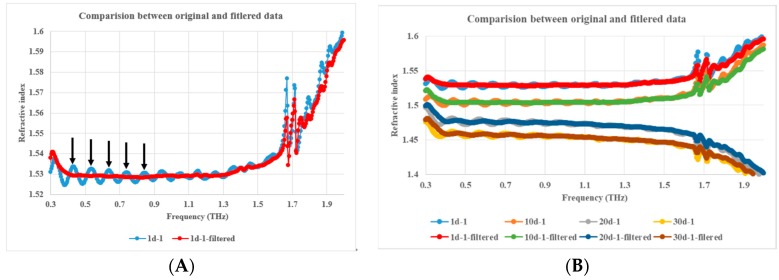
Comparison of refractive index vs. frequency between unfiltered and filtered data; (**A**). 1d-1 aged PP sample, (**B**). 1d-1, 10d-1, 20d-1, 30d-1 aged PP samples, and **A** is one part of B.

**Figure 8:**
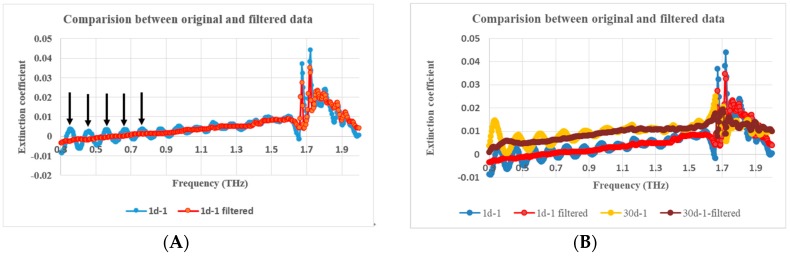
Comparison of extinction coefficient vs. frequency between unfiltered and filtered data; (**A**). 1d-1 aged PP sample, (**B**). 1d-1, 30d-1 aged PP samples, and **A** is one part of B.

**Figure 9:**
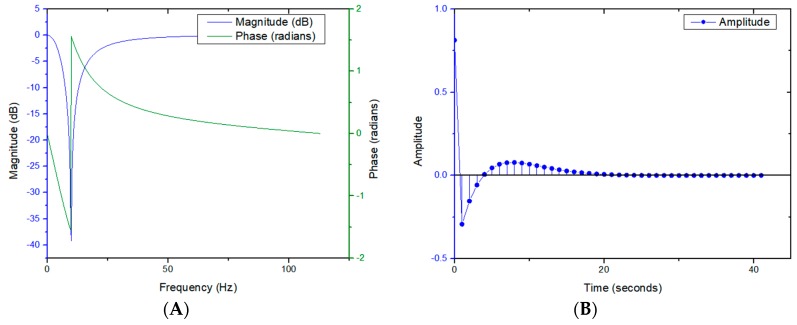
Second-order Butterworth band-stop infinite impulse response filter (IIR) filter’s response curves; (**A**). Magnitude and phase response (**B**). Impulse response.

Obviously, due to F–P resonance caused by multiple reflections at the sample surfaces when the thickness of the sample matches with the THz transmission wavelengths, the sample’s dielectric constant vs. frequency curve is superimposed with a periodic oscillation, as shown in Figure 7 and Figure 8. In order to eliminate the F–P resonance, a specifically designed second-order Butterworth band-stop infinite impulse response filter (IIR) filter was applied to process the data. The magnitude and phase response and impulse response of the filter are shown in Figure 9.

IIR filter is a kind of recursive filter, whose structure contains a feedback loop. Theoretically, its impulse response should be infinitely continuous, so its output not only depends on the current and past input signal values, but also on the past output signal values. This filter can be realized in a Butterworth, Chebyshev type-I and II, or Elliptic filter construct. Wherein, Butterworth filter has the unique characteristic that its frequency response curve in the pass band possesses the maximum flatness without fluctuation and gradually decreases to zero in the band-stop.

Obviously, the frequency data difference between every two adjacent crests indicated by the arrows is the F–P oscillation period as shown with the blue line in Figure 7A. The crests in the extinction coefficient curve with THz frequency in Figure 8A can play the same role as those in the Figure 7A, so far as F–P oscillation period identification is concerned. The reciprocal of the F–P oscillation period mentioned above is the F–P oscillation frequency in quasi space domain, which is the center frequency of the designed Butterworth band-stop IIR filter as shown as the bottom of the trough with a blue line in Figure 9A.

THz dielectric spectroscopic data of aged PP samples were processed by the above mentioned filter. The F–P resonance was eliminated to a good extent, which is validated by Figure 7A and Figure 8A. Meanwhile, the variance tendency of the dielectric constants with THz frequency was preserved, essentially unaltered, as shown in Figure 7B and Figure 8B. 

**Figure 10:**
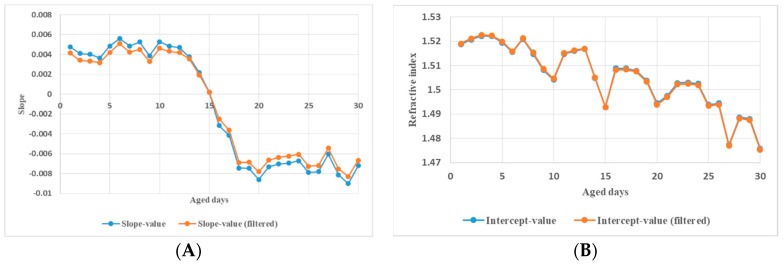
Comparison of the slope (**A**) and the intercept (**B**) of the linear fitting between unfiltered and filtered refractive index vs. frequency data.

On the other hand, in order to seek the rules between PP’s ultraviolet aging states and its dielectric spectroscopic properties, a linear fitting technique was invoked to process the dielectric constants. The relationships between the slope and the intercept of the linear fitting between refractive index and THz frequency and the aged days is shown with the blue line in Figure 10A. Basically, the early, middle, and late stages of PP ultraviolet aging can be distinguished by the slope of the linear fitting between the refractive index and THz frequency. In the whole ultraviolet aging process, the slope variation from 10-day to 20-day is the sharpest and the 15-day point is almost a zero-crossing-point (ZCP). It is inferred that the PP exhibits normal dispersion in the THz frequency band before 15-day ultraviolet aging. It then transforms into abnormal dispersion after 15-day ultraviolet aging. On the other hand, as can be seen in Figure 10B, the intercept value with aged days generally decreases with THz frequency although fluctuation exists. Such a trend with aged days is similar to that of the average refractive index in THz band (Figure 5).

**Figure 11:**
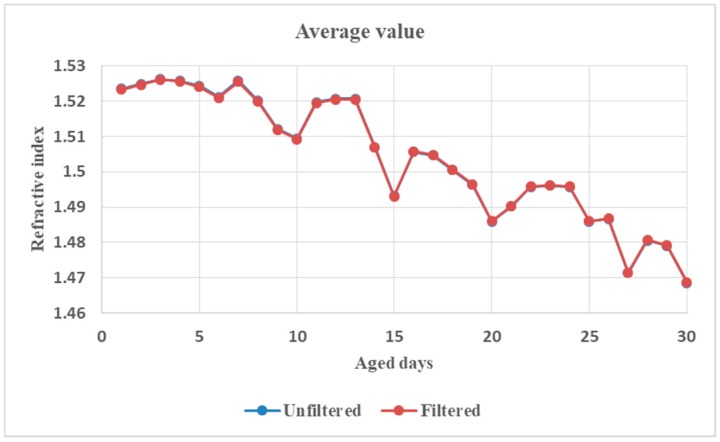
Comparison of the average value of refractive index between unfiltered and filtered data.

Besides, Figure 10 shows that the slopes and the intercepts of the linear fitting are almost identical before and after filtering. So is the average refractive index, as shown in Figure 11.

## 4. Conclusions

Based on the measurement and analysis of PP’s THz dielectric spectroscopic properties during its ultraviolet aging presented here, several key conclusions can be drawn.

**a.**  Overall, PP’s refractive index slightly decreases with its ultraviolet aging duration. It is worth noticing that PP presents normal dispersion in the THz frequency band before 15-day ultraviolet aging, that is, its refractive index increases with the increase of TH frequency. However, it transforms into abnormal dispersion after 15-day ultraviolet aging, namely, its refractive index decreases with the increase of TH frequency. This can probably be explained as due to the appearance of a THz high-frequency absorption band due to the transformation of nonpolar hydrocarbon groups to polar oxygenated groups during the process of PP ultraviolet aging. Such a band is beyond the current measurement frequency region, but its low-frequency tail certainly affects the measurement.
**b.**  Although PP material has almost no absorption to THz waves within the measurement accuracy of the THz-TDS system employed for this study, a general trend can nonetheless be inferred that the absorption slightly increases with the duration of ultraviolet aging.
**c.**  The specially designed second-order Butterworth band-stop IIR filter does a good job in eliminating the F–P resonance due to multiple reflections at the sample surfaces, without introducing any signal distortion and adversely affecting signal fidelity.
**d.**  A linear fitting method was adopted to process the data of the index of refraction as a function of THz frequency and naturally leads to an effective judging criterion of degree/length of UV aging, based on the slope value of the dielectric vs. frequency data that undergoes a drastic inflection and zero-crossing at the 15-day aging point—before this point the slope is positive and after this point the slope becomes negative. This corroborates nicely with the early-term, mid-term, and late-term phases of PP ultraviolet aging, and as such, can play the role of a unique and unambiguous marker for assessing the degree and/or length of ultraviolet aging of polypropylene.

In summary, this paper presents for the first time a full-scale, systematic examination of polymer aging due to exposure to UV radiation, using PP as a prototypical example, by THz dielectric spectroscopy. We found that the variation rate of the real part of the dielectric constant of PP changes discernibly with duration of UV exposure and exhibits an inflection point midway through the full-course aging process that can be used to delineate the early stage, mid-stage, and final stage of UV aging. We rationalize this process by considering the chemistry of oxidation induced polarization of the polymer material. This should be a useful benchmark for understanding the polymer UV aging process in general and PP in particular. In closing, we should point out that although we considered only PP in this experimental study, the lesson learned has broader ramifications for the general behavior of high-polymer materials under UV radiation, if not quantitatively, at least qualitatively.
